# Effect of Broccoli Sprouts and Live Attenuated Influenza Virus on Peripheral Blood Natural Killer Cells: A Randomized, Double-Blind Study

**DOI:** 10.1371/journal.pone.0147742

**Published:** 2016-01-28

**Authors:** Loretta Müller, Megan Meyer, Rebecca N. Bauer, Haibo Zhou, Hongtao Zhang, Shannon Jones, Carole Robinette, Terry L. Noah, Ilona Jaspers

**Affiliations:** 1 Center for Environmental Medicine, Asthma and Lung Biology, University of North Carolina at Chapel Hill, Chapel Hill, North Carolina, United States of America; 2 University Children’s Hospital Basel, Basel, Switzerland; 3 Department of Microbiology and Immunology, University of North Carolina at Chapel Hill, Chapel Hill, North Carolina, United States of America; 4 Department of Pediatric Allergy and Immunology, Stanford University, Stanford, California, United States of America; 5 Department of Biostatistics, University of North Carolina at Chapel Hill, Chapel Hill, North Carolina, United States of America; 6 Department of Pediatrics, University of North Carolina at Chapel Hill, Chapel Hill, North Carolina, United States of America; Fondazione IRCCS Ca' Granda Ospedale Maggiore Policlinico, Università degli Studi di Milano, ITALY

## Abstract

Enhancing antiviral host defense responses through nutritional supplementation would be an attractive strategy in the fight against influenza. Using inoculation with live attenuated influenza virus (LAIV) as an infection model, we have recently shown that ingestion of sulforaphane-containing broccoli sprout homogenates (BSH) reduces markers of viral load in the nose. To investigate the systemic effects of short-term BSH supplementation in the context of LAIV-inoculation, we examined peripheral blood immune cell populations in non-smoking subjects from this study, with a particular focus on NK cells. We carried out a randomized, double-blinded, placebo-controlled study measuring the effects of BSH (N = 13) or placebo (alfalfa sprout homogenate, ASH; N = 16) on peripheral blood mononuclear cell responses to a standard nasal vaccine dose of LAIV in healthy volunteers. Blood was drawn prior to (day-1) and post (day2, day21) LAIV inoculation and analyzed for neutrophils, monocytes, macrophages, T cells, NKT cells, and NK cells. In addition, NK cells were enriched, stimulated, and assessed for surface markers, intracellular markers, and cytotoxic potential by flow cytometry. Overall, LAIV significantly reduced NKT (day2 and day21) and T cell (day2) populations. LAIV decreased NK cell CD56 and CD158b expression, while significantly increasing CD16 expression and cytotoxic potential (on day2). BSH supplementation further increased LAIV-induced granzyme B production (day2) in NK cells compared to ASH and in the BSH group granzyme B levels appeared to be negatively associated with influenza RNA levels in nasal lavage fluid cells. We conclude that nasal influenza infection may induce complex changes in peripheral blood NK cell activation, and that BSH increases virus-induced peripheral blood NK cell granzyme B production, an effect that may be important for enhanced antiviral defense responses.

***Trial Registration*:** ClinicalTrials.gov NCT01269723

## Introduction

Enhancing antiviral host defense responses through nutritional supplementation would be an attractive strategy in the fight against influenza. We have recently shown that short-term ingestion of broccoli sprout homogenates (BSH) reduces markers of viral replication in nasal lavage fluid cells after inoculation with the live attenuated influenza virus (LAIV) vaccine in smokers [1]. BSH contain high levels of precursors of the antioxidant sulforaphane (SFN) [2,3], a known activator of Nrf2-dependent gene expression, which can have broad protective activities. Previous studies have shown dose-dependent, short-term effects of BSH on the expression of Nrf2-dependent enzymes in the airways [4] and in the skin [5] of human volunteers. Ingestion of broccoli sprouts has also been shown to reduce nasal allergic inflammation after diesel particle exposure [6] and to reduce air pollutant-induced toxicity [7]. In addition, we have previously shown that ingestion of BSH *in vivo*, or supplementation of epithelial cells with SFN *in vitro*, enhances antiviral host defense responses [8–10].

Besides the effects on respiratory epithelial cells, SFN can also affect immune cells including enhancing bacterial clearance by alveolar macrophages [11], augmenting the lytic activity of natural killer (NK) cells [12], and increasing overall NK cell activity in murine mouse models [13]. NK cells develop in the bone marrow and are then recruited to different sites guided by their distinct repertoire of adhesion molecules, chemokine receptors, and cell surface markers [14,15]. NK cells are crucial for innate immune responses against viruses such as influenza, [16] via cytokine release (especially interferon gamma (IFN-γ)) and cytotoxicity towards infected target cells [16]. Cytotoxic function of NK cells is marked by increased expression of the surface marker CD16, decreased expression of CD56, and increased production of lytic mediators, such as granzyme B and perforin [17]. Pathologic conditions, such as viral infections, change the microenvironment, thus driving tissue-specific differentiation of NK cells. NK cell surface receptors, such as CD314/NKG2D, recognize virus-infected target cells via the expression of ligands, such as MHC class I polypeptide-related sequence A/B or UL16 binding protein [14,15]. NK cells also express cell surface receptors allowing them to directly recognize influenza virus hemagglutinins via CD335/NKp46. To distinguish self from non-self, NK cells also express inhibitory receptors, such as CD158b/KIR2/3D and CD159a/NKG2A, which recognize human leukocyte antigen and ensure that autologous cells are only eliminated if showing strong abnormalities or signs of infection [16].

Live attenuated influenza virus (LAIV) vaccine is administered as an alternative to intramuscularly administered trivalent/quadrivalent vaccines, especially in children. Our previous studies utilizing LAIV as a model for low-level influenza infection in humans *in vivo* have demonstrated that nasal host defense responses elicited by LAIV include enhanced nasal NK cell function, a response that is blunted in smokers compared to non-smokers [18–20]. We have recently reported, in a small randomized controlled trial, that BSH can reduce markers of viral replication in nasal secretions, especially in smokers [1,18–20]. In the present study, we investigated the effects of short-term BSH supplementation in the context of LAIV inoculation on peripheral blood immune cell populations, with a particular focus on NK cells, using blood samples from non-smokers in the randomized trial. Our results show an effect of intranasal LAIV on peripheral blood T cell and natural killer T (NKT) cell populations, and on peripheral blood NK cell surface marker expression and cytotoxic activity. Additionally we demonstrate a BSH effect on NK cell granzyme B production.

## Materials and Methods

### Study design and subjects

The study was approved by the University of North Carolina (UNC) Biomedical Institutional Review Board and was registered with ClinicalTrials.gov (Identifier: NCT01269723). Written consent was obtained from each study subject prior to enrollment by the study coordinator. Consent forms were reviewed and approved by the UNC Biomedical Institutional Review Board.

**Fig 1:**
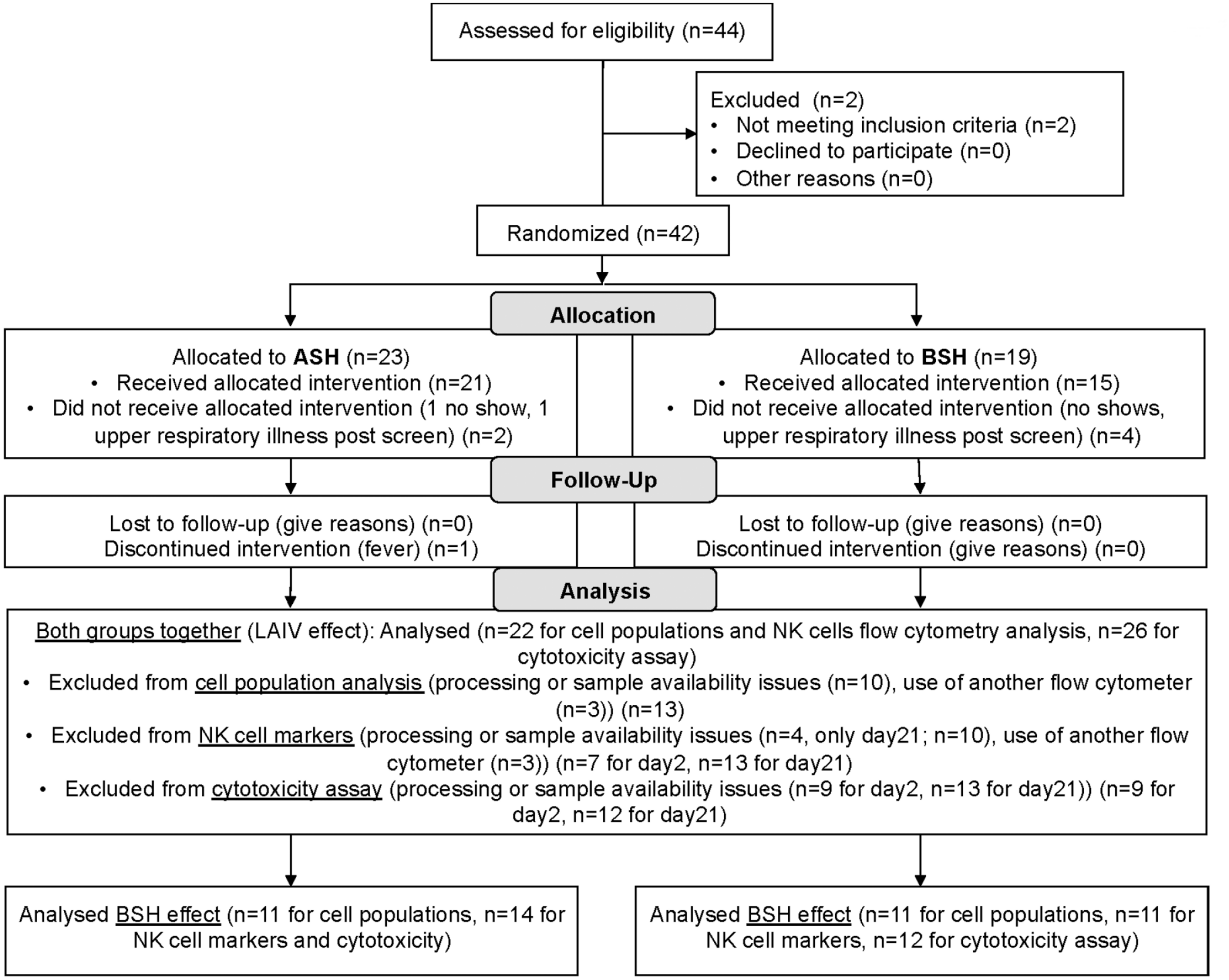
CONSORT 2010 Flow diagram for recruitment and randomization of subjects. ASH = alfalfa sprout homogenate, BSH = broccoli sprout homogenate, LAIV = life attenuated influenza virus, NK cell = natural killer cell.

We carried out a randomized, double-blind, placebo-controlled study measuring the effect of short-term ingestion of BSH on peripheral blood cell characteristics to a standard nasal vaccine dose of LAIV. Non-smoking subjects underwent screening for smoking history, informed consent, and randomization. The study design and nasal lavage fluid results have been published previously [1] (Fig 1). For the present study we only used samples from non-smoking subjects and added another blood draw to assess systemic changes at an earlier time point prior to BSH/ASH supplementation and LAIV.

**Fig 2:**
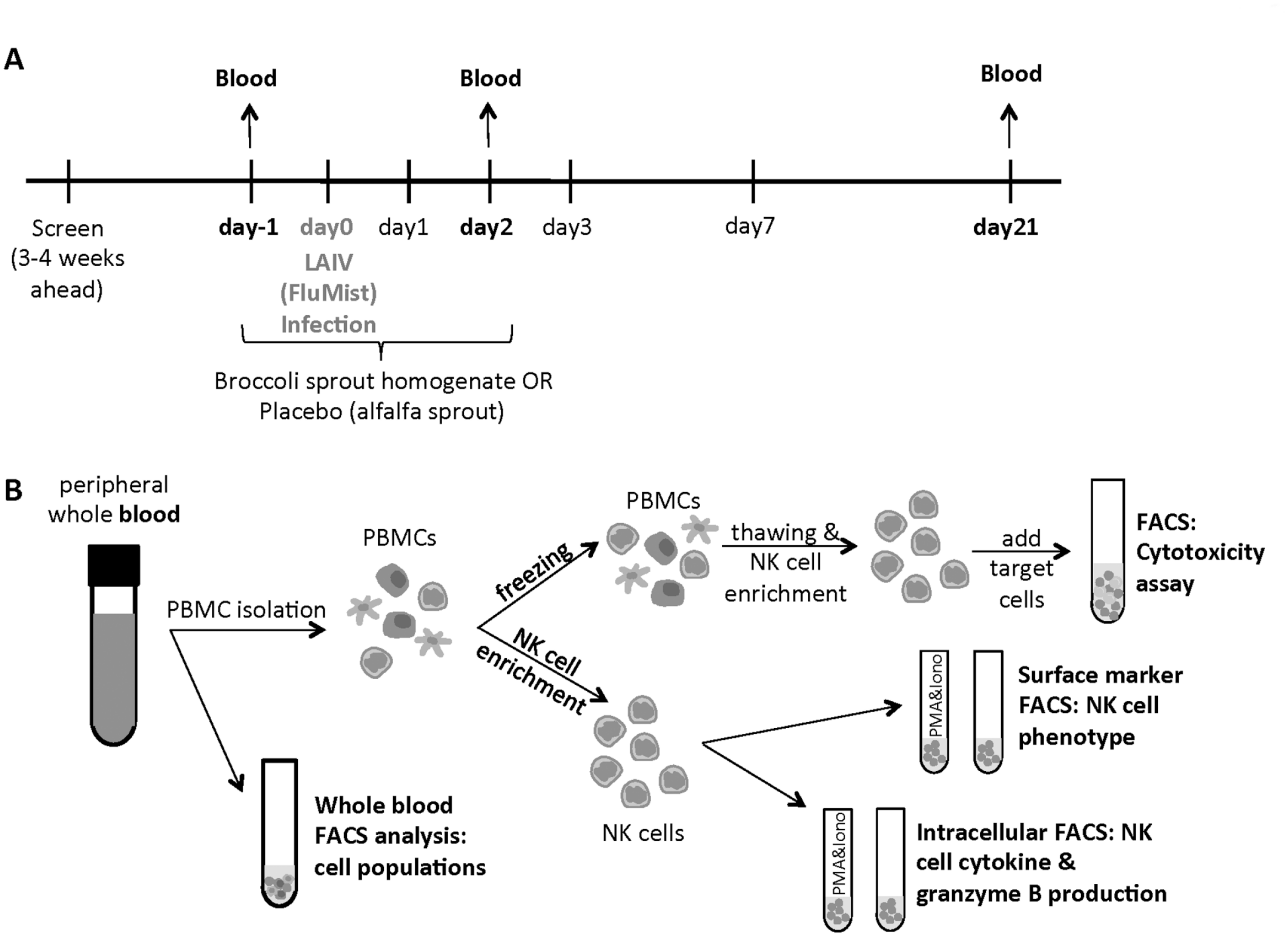
Overview of sample collection and processing. **(A)** Study design and sample collection. Details of the complete study have been published previously [1]. **(B)** Blood samples were stained for total leukocyte populations or used for NK cell enrichment. NK cells were analyzed for surface marker expression or cytokine production either naive or stimulated with PMA and ionomycin (Iono). Half of the peripheral blood mononuclear cells (PBMCs) were frozen and used later for the cytotoxicity assay.

Three to four weeks after a screening visit, subjects were randomized to receive either BSH or, as a control, alfalfa sprout homogenate (ASH). Subjects ingested daily doses of either BSH or ASH for four consecutive days, designated days-1, 0, 1, and 2 (Fig 2). ASH and BSH shakes were prepared as previously described [1,4]. Briefly, a daily portion of BSH shake was about 200g (containing about 111g of fresh broccoli sprouts (Brassica Protection Products LLC) and water). The homogenates were prepared by the clinical/translational research center’s Nutrition Research and Metabolism Core of the University of North Carolina at Chapel Hill. One dose of BSH contains about 100μmol of SFN. For the ASH, the same weight of alfalfa sprouts which contains minimal SFN was used to prepare the ASH shakes in an identical manner.

BSH or ASH was ingested by subjects under direct observation by study staff. On day0, a standard vaccine dose of LAIV (FluMist^®^, MedImmune, Inc.) was administered into each nostril according to the manufacturer’s recommendations. Peripheral blood was drawn on day-1, day2 and day21 into heparin tubes (BD Biosciences). Subjects were instructed to avoid cruciferous vegetables (which contain SFN) and anti-inflammatory medications, including corticosteroids and non-steroidal anti-inflammatory drugs, during the study period. A history of either receiving influenza vaccine or having a documented influenza infection in the previous year was a criterion for exclusion. Since the enrollment date range depended on the LAIV vaccine for the 2012–2013 influenza season, the study was conducted between September 2012 and March 2013 (exactly from October 2, 2012 to March 25, 2013).

### Whole blood flow cytometry staining

100μl whole blood was transferred to polystyrene tubes (BD Biosciences) and “Whole blood” antibody cocktail (S1 Table) was added to the samples. After 30min incubation in the dark at room temperature (RT), 2ml of 1X red blood cell lysis buffer (eBioscience, San Diego, USA) were added to each sample. After 20min of incubation (dark, RT), the samples were centrifuged (unless otherwise stated, for 10min at 500g), cell pellets were washed with 2ml phosphate-buffered saline (PBS, without Ca/Mg; Gibco, Invitrogen, Grand Island, NY, USA), centrifuged, and resuspended in 0.5ml 0.5% paraformaldehyde (PFA; Electron Microscopy Sciences, Hatfield, PA, USA) in PBS. Samples were stored in the dark at 4°C until acquisition by flow cytometry (BD LSR II with FACS Diva Software, BD Biosciences, San Jose, USA) within 24hrs. Flow cytometry data were analyzed using FlowJo Software (Ashland, OR, USA).

### PBMC isolation and, NK cell enrichment

Peripheral blood mononuclear cells (PBMCs) were isolated using a Lymphoprep^™^ (Gibco) centrifugation gradient and half of the PBMCs were used directly for NK cell enrichment as previously described [21,22]. NK cells were kept at a concentration of 10^6^ NK cells/ml in media (RPMI-1640 with L-Glutamine (Gibco) with 10% heat-inactivated fetal bovine serum (FBS; Gibco) and 1% Penicillin/Streptomycin (Gibco)) until analysis. The other half of the PBMCs was frozen based on a method by Paich et. al. [23]).

### NK cell treatment

NK cells were either analyzed directly after the enrichment or were stimulated for 4hrs with 50ng/ml phorbol 12-myristate 13-acetate (PMA; Acros Organics, Fisher Scientific) and 1μg/ml ionomycin (MP Biomedicals). For intracellular staining of cytokines and granzyme B, the Golgi block brefeldin A (eBioscience) was added.

### NK cell flow cytometry staining

NK cells were washed with flow cytometry staining buffer (PBS without Ca^2+^ and Mg^2+^, 1% heat-inactivated FBS and 0.09% sodium azide (Sigma)) and stained for surface markers with the appropriate antibody cocktail (S1 Table). After washing with 1ml flow staining buffer, cells were either fixed in 0.3ml 0.5% PFA in PBS and stored in the dark at 4°C (surface maker staining tubes) or stained for intracellular markers with the BD Cytofix/Cytoperm^™^ kit following supplier’s instructions and using the appropriate antibody cocktails. All samples were analyzed within 24hrs on the BD LSR II flow cytometer.

### NK cell cytotoxicity assay

PBMCs were thawed quickly in a 37°C water bath and 1ml of warm NK cell media was added dropwise over a 30-second period. The cell suspension was transferred to a 15ml conical tube with 8ml of warm media and centrifuged (250g, 5min, RT). After aspirating the supernatant and resuspension in media, NK cell enrichment was performed as described above.

After NK cell enrichment the 7-AAD/CFSE Cell-Mediated Cytotoxicity Assay Kit (Cayman Chemical Company, Ann Arbor, MI, USA) was performed as indicated by the manufacturer. We incubated 200,000 NK cells/tube with the human erythromyeloblastoid leukemia cell line K562 as target cells (stained previously with carboxyfluorescein succinimidyl ester) at a ratio of NK:target cells = 5:1 for 4hrs at 37°C. After viability staining with 7-aminoactinomycin D, samples were measured immediately using the BD LSR II flow cytometer.

### Quantification of nasal viral load

Markers of LAIV quantity were measured in cells of nasal lavages via quantitative real-time reverse transcription polymerase chain reaction (RT-PCR) as described by us before [1].

### Statistical analysis

For assessment of LAIV effects, we initially analyzed the subjects of the ASH and BSH groups together because most endpoints trended in the same direction. We compared the post-vaccination endpoints of day2 and day21 to the baseline on day-1 using a paired t-test. In addition, we analyzed the BSH and ASH groups separately because for some endpoints the results trended in different directions and thus appeared to be driven by one group. We compared the responses of these groups using the two sample Student’s t-test. Correlation between nasal viral load (log transformed to ensure normality) and granzyme B in blood NK cells was examined using the Pearson correlation. In all, P<0.05 was considered statistically significant. The analysis was done using the SAS program.

The sample size of the study described here was based on subjects enrolled in a previously published study [1]. For this previous study, the target sample size was derived by estimating a significant effect of BSH on LAIV-induced nasal lavage IL-6 levels using data from another previously published study [20]. Based on these data the minimum sample size to detect a treatment effect of 150% was estimated to be about 17 subjects per treatment group. Of the 35 nonsmokers who were included in the previously published study [1], we analyzed peripheral blood samples from a subset of 29 subjects. Reduced sample size was caused by change in flow cytometer parameters and antibody cocktails, missing study days, and failure to isolate sufficient PBMCs in some samples (Fig 1). All sample analysis and sample exclusion was done blinded without knowing the randomization key for treatment.

The two treatment groups were compared using a two sample t test comparing means (for age and BMI) and two sample Z test comparing proportions (for gender and race).

## Results

### Subject characteristics

Subjects were studied during the 2012–13 vaccine season and thus received LAIV containing the following influenza strains: A/California/7/2009-like (pH1N1), A/Victoria/361/2011-like (H3N2), and B/Wisconsin/1/2010-like. No subject reported intolerable taste or side effects from the shakes. One subject fainted during the blood draw on day21. There were no statistically significant differences regarding age, gender, race or BMI between the BSH and ASH treatment groups, although there was a higher proportion of females in the ASH group (Table 1). Study flow diagrams for the phases of recruitment, allocation, and data analysis are shown in Fig 1.

**Table 1:** Demographic characteristics of subjects included in this study.

Treatment	Age (year)^1^	Gender^2^	BMI^1^	Race^3^
ASH (N = 16)	27.6±1.5	12/4	25.1±1.0	12/4/0
BSH (N = 13)	25.5±1.5	7/6	25.5±1.1	9/2/2
**Total (N = 29)**	**26.7±1.1**	**19/10**	**25.3±0.7**	**21/6/2**

^1^ Mean ± standard error,

^2^ Female/male,

^3^ White/African American/Asian

### LAIV and BSH effects on peripheral blood cell populations

We measured the cell populations in the peripheral blood on day-1, day2 and day21 by flow cytometry. S1 Fig shows the gating strategy we applied for the identification of the peripheral cell populations and Table 2 summarizes the relative sizes of the cell populations regardless of the treatment group. T cell and NKT cell populations were slightly but statistically significantly reduced on day2 and day21 (only NKT cells) after intranasal LAIV-inoculation. We did not find any significant changes after LAIV for neutrophils, NK cells, monocytes or macrophages.

**Table 2:** LAIV effect on cell populations in the peripheral blood (regardless of treatment).

Cell type	day-1	day2	p value (day 2 vs. day-1)	day21	p value (day 21 vs day-1)
Neutrophils	54.3±12.1	58.2±7.28	0.12	56.4±5.93	0.46
T cells	26.5±8.07	22.7±5.27	0.022*	26.0±6.29	0.77
NKT Cells	1.65±1.15	0.93±0.61	0.002**	1.23±0.86	0.036*
NK Cells	7.38±3.5	7.12±2.5	0.72	6.85±2.1	0.44
Monocytes	52.4±12.6	50.7±12.0	0.55	48.4±12.4	0.29
Macrophages	13.7±9.25	13.2±5.57	0.79	12.6±4.25	0.59

Blood was drawn on day-1, day2 and day21 and analyzed for leukocyte populations using flow cytometry. Percentages of cells identified as neutrophils, T cells, NKT cells, NK cells, monocytes and macrophages out of all CD45^+^ cells are shown. Data are presented as mean±standard deviation (std.dev.), N = 22,

* significantly different from day-1 (p<0.05) or

** (p<0.01), tested with paired t test.

BSH supplementation resulted in a significant reduction of the NKT cell population in peripheral blood cells compared to the ASH treatment on day21, but not on day2 (Table 3). None of the other cell populations were affected by BSH supplementation, including examining the ratio of day21 or day2 to day-1 (S2 Table).

**Table 3:** BSH effect on cell populations in the peripheral blood.

Cell type	Difference day2 minus day-1			Difference day21 minus day-1		
	ASH	BSH	p value	ASH	BSH	p value
Neutrophils	2.79±11.1	5.02±11.1	0.65	1.39±10.0	2.89±16.0	0.80
T cells	-3.56±7.92	-4.23±7.20	0.84	0.209±6.00	-1.27±10.6	0.69
NKT Cells	0.742±0.95	0.705±1.02	0.93	0.037±0.55	0.804±1.00	0.041*
NK Cells	0.357±3.06	0.155±3.69	0.89	0.217±2.45	0.829±3.78	0.66
Monocytes	0.882±14.8	-2.69±13.1	0.77	0.200±13.7	-7.91±20.4	0.31
Macrophages	0.725±5.01	0.291±11.5	0.91	-1.58±4.71	0.655±13.1	0.83

Blood was drawn on day-1, day2 and day21 and analyzed for leukocyte populations using flow cytometry. Percentages of cells identified as neutrophils, T cells, NKT cells, NK cells, monocytes and macrophages out of all CD45+ cells were identified and differences of day2 or day21 and day-1 are shown. Data are presented as mean±std.dev., N = 10–11.

* significantly different (p<0.05), tested with two sample t test.

### LAIV and BSH effect on peripheral NK cell function

**Fig 3:**
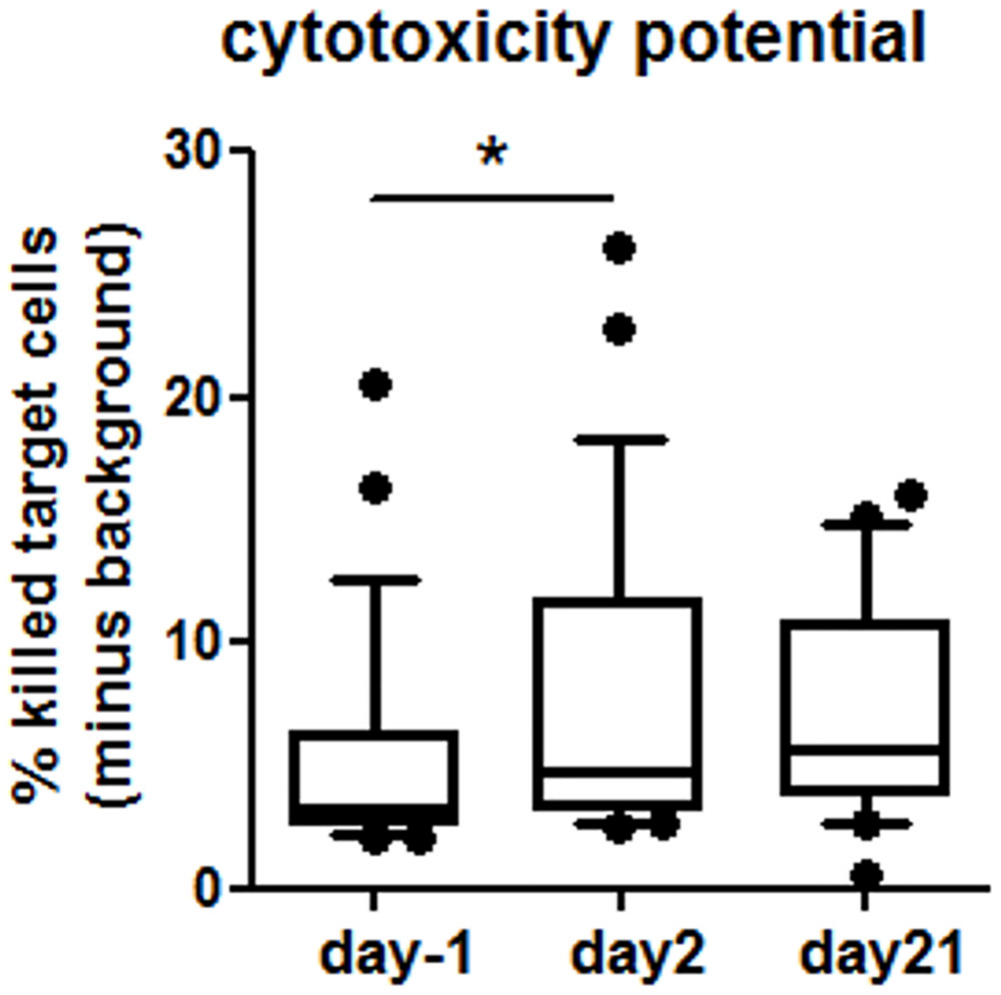
LAIV effect on cytotoxicity potential of systemic NK cells (regardless of treatment). Following NK cell enrichment, NK cells were incubated with K562 target cells for 4hrs and the cell mixture was stained for viability. N = 26 (day-1 and 2), N = 22 (day21). Data are shown as whiskers with 10–90 percentiles. *significantly different (p = 0.015), tested with paired t test.

NK cells did not show many LAIV-induced effects in surface marker expression or intracellular mediator expression in an unstimulated setting (data not shown), but did display changes upon further stimulation with PMA and ionomycin. LAIV enhanced markers of peripheral blood NK cell activation on day2. Both mean fluorescence intensity (MFI) and percentage of cells expressing the inhibitory NK cell receptor CD158b were significantly reduced, and the expression of the cytotoxic NK cell receptor CD16 was increased (Table 4; S3 Table). The overall MFI of CD56 expression on NK cells was also significantly reduced, suggesting increased presence of CD56^dim^ NK cells, which are more cytotoxic. We did not find any changes in the expression of the CXCL10/IP-10 receptor CXCR3/CD183 or the activation receptor CD314 when focusing on MFI. However, on day2 the percentage of CD314^+^ cells and on day21 the percentage of CD183^+^ cells were significantly reduced compared to day-1. A significant reduction of the percentage of CD56^+^ cells was also found on day21 (S3 Table). Nasal LAIV did not change intracellular expression of IFN-γ, IL-4 or granzyme B in NK cells (Table 4; S3 Table). However, the potential of peripheral NK cells to kill target cells on day2 was significantly increased by intranasal administration of LAIV (Fig 3), suggesting that the LAIV-induced increase of peripheral blood CD56^dim^CD16^+^ NK cell population at day2 functionally reflects the increased cytotoxic potential of these cells.

**Table 4:** LAIV effect on markers of systemic NK cells (regardless of treatment).

Marker	day-1	day2	p value (day 2 vs. day-1)	day21	p value (day 21 vs day-1)
CD56	1297±709	1061±513	0.0084*	1053±733	0.19
CD16	6233±5808	8741±5193	0.0095*	5434±5112	0.52
CD314 (NKG2D)	419±131	371±118	0.05	411±123	0.72
CD158b	995±522	667±297	0.0007***	886±643	0.33
CD183 (CXCR3)	3385±7362	710±363	0.11	1226±1120	0.14
IFN-γ	474±424	457±304	0.74	499±462	0.84
IL-4	430±176	435±232	0.96	427±196	1.00
Granzyme B	1528±630	1440±939	0.64	1514±904	0.73

Following NK cell enrichment, NK cells were stimulated with PMA/Ionomycin and blocked with Brefeldin A (only intracellular markers) for 4hrs. Data are presented as mean±std.dev. of MFI. N = 22–28.

* significantly different from day-1 (p<0.05) or

*** (p<0.001), tested with paired t test.

**Fig 4:**
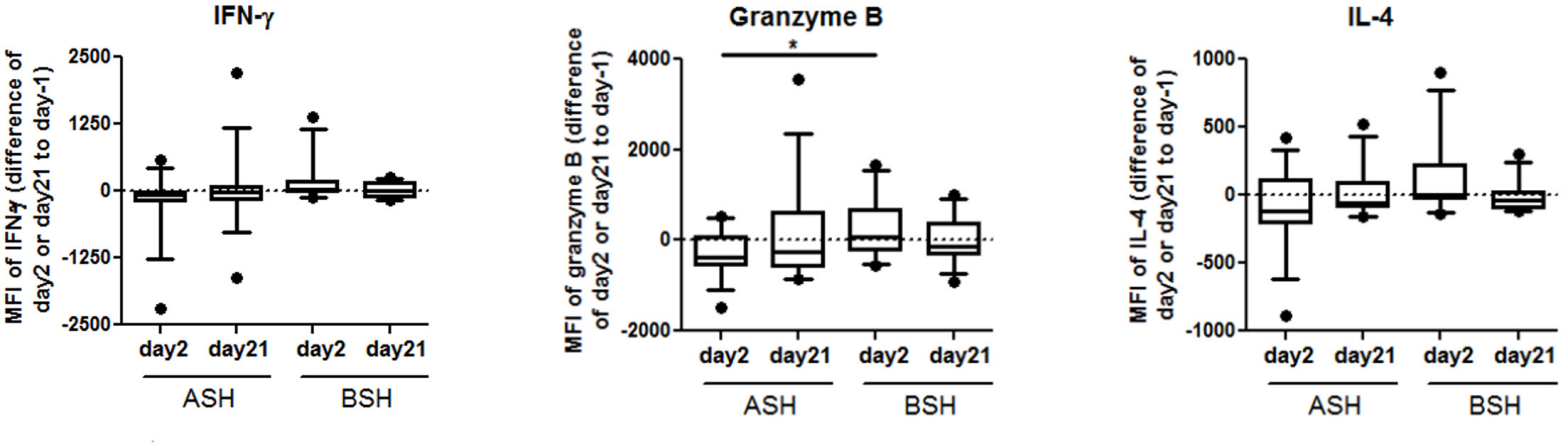
BSH effect on intracellular markers of systemic NK cells. Following NK cell enrichment, NK cells were stimulated with PMA/Ionomycin and blocked with Brefeldin A for 4hrs. The differences of day2 or day21 and day-1 are shown. Data are presented as mean±std.dev. N = 9–14. Data are shown as whiskers with 10–90 percentiles. *significantly different (p = 0.049), tested with two sample t test.

None of the NK cell surface markers differed between the BSH and ASH groups on day2 (Table 5, S4 Table). On day21, CD314 was increased in the BSH group, but decreased in the placebo group as compared to day-1, both when comparing the percentage of cells expressing CD314 and MFI (Table 5, S4 Table). None of the other surface markers changed on day21. To determine potential effects of SFN in the BSH on NK cell mediator production, we compared the levels of intracellular granzyme B, IL-4, and IFN-γ in subjects receiving ASH and BSH supplementation. As compared to day-1, granzyme B expression was increased on day2 in the BSH group and was also significantly different from the ASH group. We found similar trends for IL-4 and IFN-γ, albeit not statistically significant (Fig 4). The potential of peripheral NK cells to kill target cells was not different between the ASH and BSH groups (S2 Fig).

**Table 5:** BSH effect on markers of systemic NK cells.

Marker	Difference day2 minus day-1			Difference day21 minus day-1		
	ASH	BSH	p value	ASH	BSH	p value
CD56	-447±540	-187±564	0.28	-433±957	-42.9±929	0.30
CD16	3792±5519	2083±4728	0.43	-1401±7858	-353.3±6378	0.71
CD314 (NKG2D)	-90.6±135	-26.8±163	0.33	-53.6±111	44.5±107	0.038*
CD158b	-316±365	-185±236	0.31	-50.2±771	-203±375	0.52
CD183 (CXCR3)	-2379±6931	-3497±9024	0.76	-2041±6191	-2854±8176	0.81

Following NK cell enrichment, NK cells were stimulated with PMA/Ionomycin and blocked with Brefeldin A (only intracellular markers) for 4hrs. The differences of day2 or day21 and day-1 are shown. Data are presented as mean±std.dev. N = 9–14.

* significantly different (p<0.05), tested with two sample t test.

### Correlation between nasal viral load and granzyme B in peripheral NK cells

**Fig 5:**
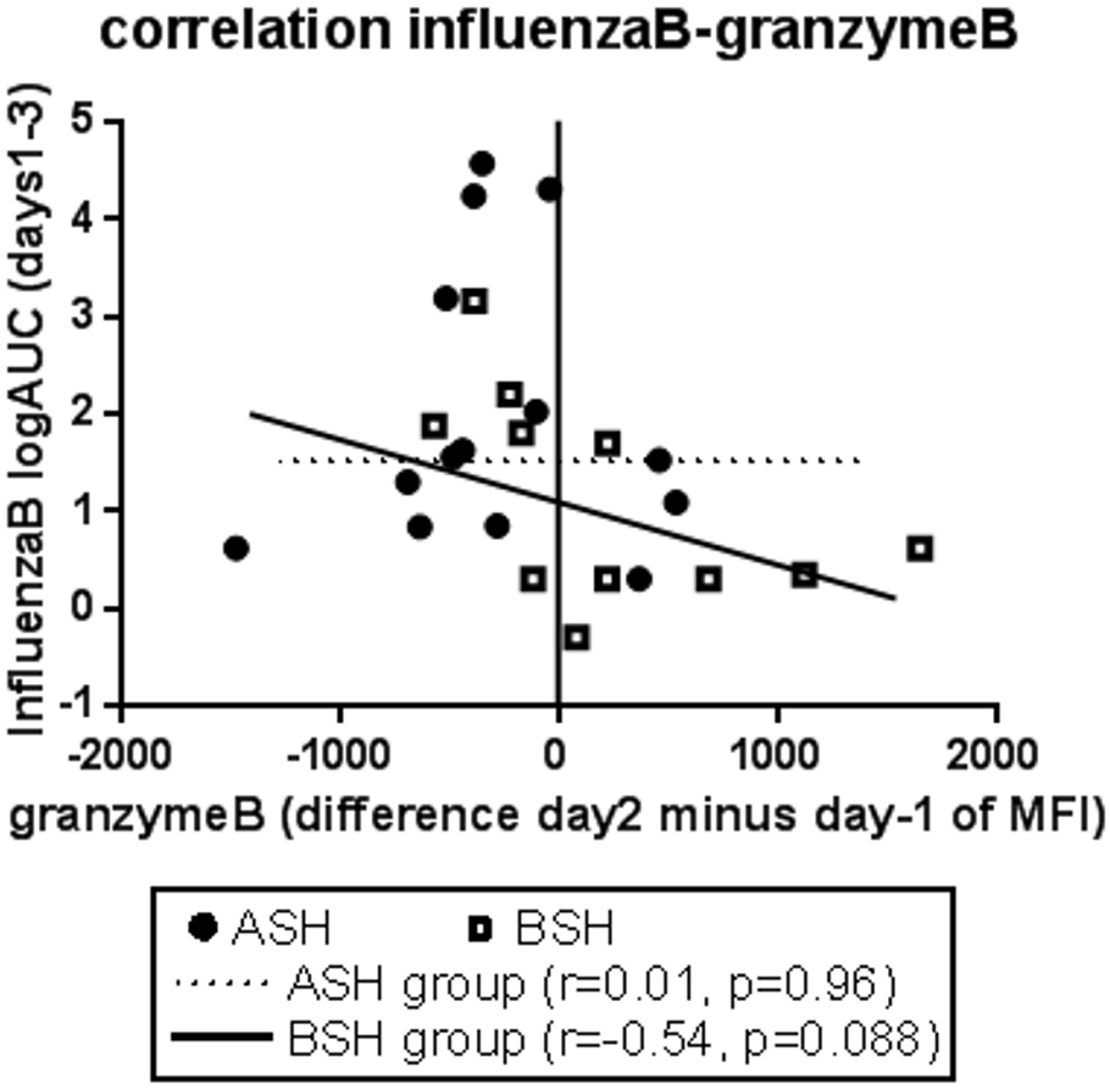
Correlation between nasal influenza B virus load and granzyme B in systemic NK cells. Viral load in nasal lavage fluid cells collected after inoculation with LAIV was detected via RT-PCR to Flu B RNA and expressed as log transformed area under the curve (details see [1]). The correlation with granzyme B levels in systemic NK cells was tested with the Pearson correlation for both groups.

In the BSH, but not the ASH group, granzyme B levels in peripheral NK cells appeared to have a negative relationship with LAIV-specific Influenza RNA levels in nasal lavage fluid cells, albeit not statistically significant (p = 0.088) (Fig 5).

## Discussion

We investigated peripheral blood immune cell activation in the context of a randomized, double-blinded, placebo-controlled study measuring the effects of SFN-rich BSH on responses to a standard nasal vaccine dose of LAIV. Independently of BSH or ASH supplementation, intranasal LAIV infection itself slightly reduced the relative sizes of T cell and NKT cell populations in peripheral blood. In NK cells, LAIV increased killing potential and CD16 expression, and reduced the expression of CD56 and CD158b. Compared to ASH, BSH decreased the size of the NKT cell population. BSH also increased CD314 expression on day21, and increased granzyme B production by peripheral NK cells on day2. The granzyme B levels in peripheral NK cells In the BSH, but not the ASH group, appeared to be negatively associated with Influenza RNA levels in nasal lavage fluid cells, which may have played a role in reducing replication of LAIV in the nasal cavity.

Granzyme B production is important for NK cells’ ability to kill target cells [16]. We have previously demonstrated that decreased nasal granzyme B levels coincided with decreased cytotoxic NK cell populations and increased markers of viral replication in smokers inoculated with LAIV [1,18,19]. In addition, we demonstrated that BSH supplementation decreased markers of viral replication in smokers and nonsmokers [1]. The data shown here indicate that the SFN of the BSH supplementation increases granzyme B levels in peripheral blood NK cells in nonsmoking individuals and that these systemic effects may be inversely related to viral load in nasal lavage fluid cells, suggesting that SFN-induced effects on peripheral blood NK cells could affect antiviral host defense responses in the nasal mucosa. SFN has been shown to increase cytotoxicity of NK cells in the context of prostate carcinogenesis inhibition in mice [12], to augment NK cells cytotoxicity in tumor-bearing BALB/c mice [13] and to induce NKG2D ligands in human cancer cell lines and thus enhance susceptibility of cancer cells to NK cell mediated lysis [24]. However, we did not find a SFN-induced increase of the cytotoxic potential of NK cells beyond the effects of LAIV. Possible reasons include the strong LAIV-and sprout-induced increase of the cytotoxic potential in both treatment groups on day2 masking any potential SFN effect. In addition, the cytotoxicity assay, which assessed the killing of target tumor cells, may not accurately reflect cytotoxicity towards influenza-infected cells, which is mediated by additional natural cytotoxicity receptors expressed on NK cells, such as CD335/NKp46 and NKp44 recognizing influenza hemagglutinins [25].

Other studies investigating BSH effects, and more specifically SFN, show a broad spectrum of SFN effects with regards to antiviral function. SFN has been shown to inhibit the lytic cycle of the Epstein-Barr virus and prevent virus reactivation [26]. Pretreatment with SFN limited lung respiratory syncytial virus replication and virus-induced inflammation in mice [27]. We have previously demonstrated that SFN reduces susceptibility to influenza infections by inhibiting viral entry [9]. Furthermore, rodent studies suggested that SFN-induced activation of the Nrf2/antioxidant response element pathway could augment antioxidant defenses and improve lung health in HIV-infected individuals [28]. Our data adds to the mounting literature demonstrating that SFN has broad antiviral potential, and shows that in human volunteers SFN supplementation may alter the course of influenza viral infection.

We found a decrease in peripheral blood NKT and T cell populations on day2 post inoculation, and in NKT cells on day21, but not in total NK cells or other studied cell populations (such as monocytes, macrophages or neutrophils). This is in accordance with the results of our previous study, which did not find any changes of total NK cell or neutrophil population sizes in the nose after LAIV [19]. Several studies have investigated the effect of influenza vaccines on T cell populations, demonstrating that LAIV increases peripheral blood memory T cells in children, but decreases CD8^+^CD27^+^ T cells in adults 10 days post inoculation [29–31]. Rudenko et al. [32] found an increase of antigen-specific CD4^+^ and CD8^+^ memory T cells after two-dose vaccination with H7N3 avian flu LAIV. Since we only examined total T cells, we cannot further identify whether the LAIV-induced reduction in the T cell population was limited to CD8^+^ cells or also included other T cell subsets. In addition, our data indicate a decrease in peripheral blood T cell populations much earlier on day2 post-inoculation. We have previously shown that LAIV-induced T cell recruitment to the nasal mucosa occurs within four days post inoculation [18], suggesting that the drop in peripheral blood T lymphocytes may be caused by recruitment of these cells to the nasal mucosa. Similarly, we hypothesize that the decrease in LAIV-induced peripheral blood NKT cells represents migration to the infection locations. NKT cells rapidly produce cytokines upon activation, e.g. by microbial stimuli, yet their exact role in fighting influenza infections and their potential role in the response to influenza vaccination remain unclear. Thus, we believe that the changes in peripheral blood T cell and NKT cell populations could be a result of cell trafficking towards the site of LAIV infection.

We have previously shown an increase in CD16^+^ NK cells and granzyme B levels in the nose in response to LAIV, and that this effect was suppressed in smokers [19]. Whether this change in nasal NK cell population was caused by smoking-induced changes in the nasal microenvironment leading to modulation of local NK cell maturation or systemic alteration of this cell population is unclear. In the present study we examined the *in vivo* LAIV effect on peripheral NK cells of nonsmoking subjects and found an increase in NK cell activation (increased CD16, decreased CD158b, increased cytotoxic potential). NK cells showed a decrease, albeit not statistically significant, of the activation marker CD314 expression on day2. This effect may be due to the recruitment of NK cells with high CD314 expression to the infection site and thus a decrease of this cell population in peripheral blood. LAIV-induced effects on systemic NK cell populations have been reported previously. Dou et al. [33] showed increased IFN-γ production of NK cells in the first 3 months after LAIV vaccination and restimulation with influenza virus *in vitro*, leading to the hypothesis that this could be an “intracellular immune memory of human NK cells” playing an important role for the influenza vaccination. Our data together with previous studies suggest an important role for LAIV-induced systemic effects on NK cells, which may contribute to the short- and long-term roles of NK cells in the context of a viral infection.

All participants received a LAIV dose and nutritional supplementation (either ASH or BSH), thus we did not include groups without nutritional supplementation or without LAIV-infection. This allows us to compare specifically the effect of the SFN, which is only contained in BSH, but not in ASH, on LAIV-infection. However, we did not investigate the effects of nutritional supplementation alone or the effects of the sprout components in the supplementation. For example, bacterial components of sprouts have been shown to be protective against influenza infection in mice [34], to increase the activity of macrophages *in vitro* [35] and to enhance NK cell activity in healthy human volunteers [36]

Our data show further enhancement of LAIV-induced granzyme B production by NK cells in subjects supplemented with SFN containing BSH. There was a tendency for the SFN-induced increase in granzyme B production in peripheral blood NK cells to be inversely related to markers of viral load in the nose, suggesting a potential mechanism by which systemic effects of BSH supplementation, and more specifically of the SFN contained in BSH, can be manifested at respiratory mucosal sites of infection. It is not clear whether similar effects of SFN supplementation can be observed in nasal mucosal NK cells and what the cellular mechanisms are by which BSH or SFN modify NK cell function. Further studies are needed to see whether BSH supplementation directly enhances peripheral blood NK cell function, as supported by previous *in vitro* studies [12] or whether this is an indirect effect on the expression of ligands or cytokines known to enhance granzyme B production. Taken together, our data sheds insight on the effects of LAIV on systemic immune cells and supports continued investigation into understanding how specific nutritional supplementation can enhance respiratory antiviral defense responses.

## Supporting Information

S1 CONSORT Checklist CONSORT Checklist. (DOC)

S1 Fig Gating strategy for peripheral blood cell populations. After gating on CD45+ cells, we gated on specific markers for neutrophils (CD66b+), T cells (CD3+), NK cells (CD56+CD66b-CD3-), NKT cells (CD56+CD66b-CD3+), monocytes (CD14dim) and macrophages (CD14bright).
(DOCX)

S2 Fig BSH effect on cytotoxicity potential of systemic NK cells. Following NK cell enrichment, NK cells were incubated with K562 target cells and stained for viability. Data are shown as mean±std.dev. of the difference between day2 or day21 and day-1. N = 15 (day2 minus day-1), N = 12 (day21 minus day-1).
(DOCX)

S1 Table Antibody cocktails used for flow cytometry staining. (DOCX)

S2 Table BSH effect on cell populations in the peripheral whole blood (fold induction of day-1). Blood was drawn on day-1, day2 and day21 and analyzed for leukocyte populations using flow cytometry. The ratios of day2 or day21 to day-1 are shown. Data are presented as mean±std.dev., N = 10–11.
(DOCX)

S3 Table LAIV effect on markers of systemic NK cells (percentage of positive cells; regardless of treatment). Following NK cell enrichment, NK cells were stimulated with PMA/Ionomycin and blocked with Brefeldin A (only intracellular markers) for 4hrs. Data are presented as mean±std.dev. of percentage of positive cells. N = 22–29. *significantly different from day-1 (p<0.05), tested with paired t test.
(DOCX)

S4 Table BSH effect on markers of systemic NK cells (fold induction of day-1). Following NK cell enrichment, NK cells were stimulated with PMA/Ionomycin and blocked with Brefeldin A (only intracellular markers) for 4hrs. The ratio of day2 or day21 to day-1 are shown. Data are presented as mean±std.dev. N = 9–14. *significantly different (p<0.05), tested with two sample t test.
(DOCX)
