# Supplementary material for: Effect of Broccoli Sprouts and Live Attenuated Influenza Virus on Peripheral Blood Natural Killer Cells: A Randomized, Double-Blind Study
Source: PLoS One. 2016 Jan 28;11(1):e0147742. doi: 10.1371/journal.pone.0147742 (PMC4731143; doi:10.1371/journal.pone.0147742)

S1 Figure . Gating strategy for peripheral blood cell populations. After gating on CD45+ cells, we gated on specific markers for neutrophils (CD66b+), T cells (CD3+), NK cells (CD56+CD66b-CD3-), NKT cells (CD56+CD66b-CD3+), monocytes (CD14dim) and macrophages (CD14bright).


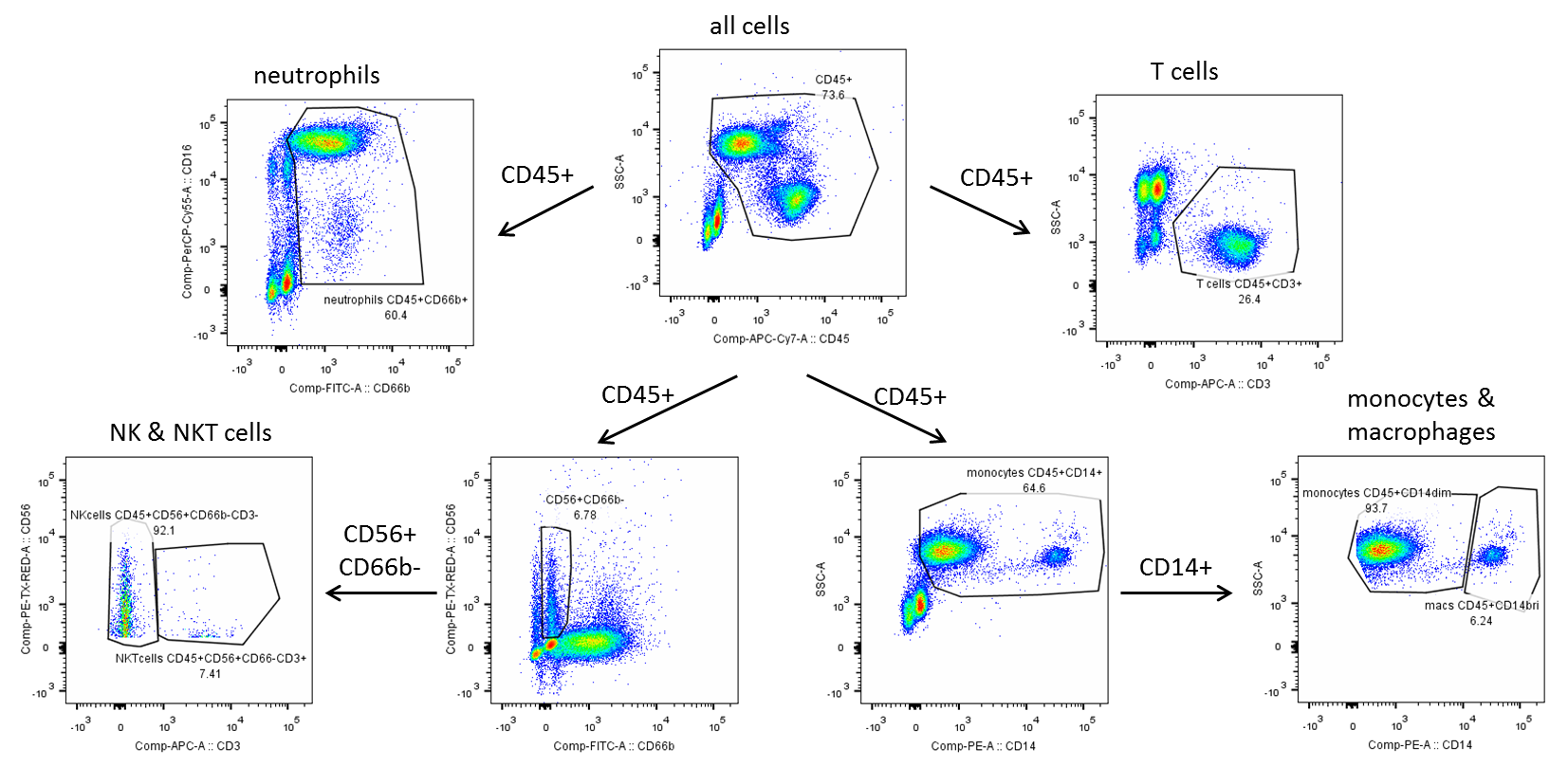

Supplement: S1 Fig — After gating on CD45+ cells, we gated on specific markers for neutrophils (CD66b+), T cells (CD3+), NK cells (CD56+CD66b-CD3-), NKT cells (CD56+CD66b-CD3+), monocytes (CD14dim) and macrophages (CD14bright). (DOCX) [file pone.0147742.s002.docx]
