# Supplementary material for: Effect of Broccoli Sprouts and Live Attenuated Influenza Virus on Peripheral Blood Natural Killer Cells: A Randomized, Double-Blind Study
Source: PLoS One. 2016 Jan 28;11(1):e0147742. doi: 10.1371/journal.pone.0147742 (PMC4731143; doi:10.1371/journal.pone.0147742)
Supplement: S2 Fig — Following NK cell enrichment, NK cells were incubated with K562 target cells and stained for viability. Data are shown as mean±std.dev. of the difference between day2 or day21 and day-1. N = 15 (day2 minus day-1), N = 12 (day21 minus day-1). (DOCX) [file pone.0147742.s003.docx]

S2 Figure. BSH effect on cytotoxicity potential of systemic NK cells. Following NK cell enrichment, NK cells were incubated with K562 target cells and stained for viability. Data are shown as mean±std.dev. of the difference between day2 or day21 and day-1. N=15 (day2 minus day-1), N=12 (day21 minus day-1).
