# Supplementary material for: Effect of Broccoli Sprouts and Live Attenuated Influenza Virus on Peripheral Blood Natural Killer Cells: A Randomized, Double-Blind Study
Source: PLoS One. 2016 Jan 28;11(1):e0147742. doi: 10.1371/journal.pone.0147742 (PMC4731143; doi:10.1371/journal.pone.0147742)
Supplement: S1 Table — (DOCX) [file pone.0147742.s004.docx]

S1 Table. Antibody cocktails used for flow cytometry staining

| **Antibody cocktail** | **FITC** | **APC** | **PE** | **PE-TR** | **PerCP-Cy5.5** | **APC-Cy7** |
| --- | --- | --- | --- | --- | --- | --- |
| Whole blood samples | 2.5µl CD66b | 5µl CD3 | 1.25µl CD14 | 2.5µl CD56 | 5µl CD16 | 2.5µl CD45 |
| Whole blood **isotype** control | 0.25µl IgG1 | 0.5µl IgG1 | 0.125µl IgG2 | 0.25µl IgG2 | 0.5µl IgG1 | 2.5µl CD45 |
| NK cell surface marker samples | 5µl CD16 | 5µl CD314 (NKG2D) | 2.5µl CD158b | 2.5µl CD56 | 1.25µl CD183 (CXCR3) | - |
| NK cell surface marker **isotype** control | 0.5µl IgG1 | 0.5µl IgG1 | 0.25µl IgG2 | 2.5µl IgG2 | 0.125µl IgG1 | - |
| NK cell intracellular samples | 5µl Granzyme B* | 0.625µl IFN-γ* | 5µl IL-4* | 2.5µl CD56 | 5µl CD16 | 5µl CD3 |
| NK cell intracellular **isotype** control | 0.5µl IgG1* | 0.063µl IgG1* | 0.5µl IgG1* | 0.25µl IgG2 | 0.5µl IgG1 | 0.5µl IgG1 |

*Used during the intracellular (second) staining step.

Antibodies were purchased from BD Biosciences (CD3-APC, CD16-PerCP/Cy5.5, CD158, CD45, Granzyme B), Biolegend (CD183, CD3-APC/Cy7, CD314), eBioscience (IFN-γ, IL-4, CD14) Beckman Coulter (CD66b, CD16-FITC) and Invitrogen (CD56).
