# Supplementary material for: Effect of Broccoli Sprouts and Live Attenuated Influenza Virus on Peripheral Blood Natural Killer Cells: A Randomized, Double-Blind Study
Source: PLoS One. 2016 Jan 28;11(1):e0147742. doi: 10.1371/journal.pone.0147742 (PMC4731143; doi:10.1371/journal.pone.0147742)
Supplement: S2 Table — Blood was drawn on day-1, day2 and day21 and analyzed for leukocyte populations using flow cytometry. The ratios of day2 or day21 to day-1 are shown. Data are presented as mean±std.dev., N = 10–11. (DOCX) [file pone.0147742.s005.docx]

S2 Table. BSH effect on cell populations in the peripheral whole blood (fold induction of day-1). Blood was drawn on day-1, day2 and day21 and analyzed for leukocyte populations using flow cytometry. The ratios of day2 or day21 to day-1 are shown. Data are presented as mean±std.dev., N=10-11.

| **Cell type** | **Ratio of day2/day-1** | | | **Ratio of day21/day-1** | | |
| --- | --- | --- | --- | --- | --- | --- |
|  | **ASH** | **BSH** | **p value** | **ASH** | **BSH** | **p value** |
| Neutrophils | 1.09±0.22 | 1.16±0.35 | 0.59 | 1.06±0.22 | 1.14±0.44 | 0.62 |
| T cells | 0.937±0.37 | 0.921±0.36 | 0.92 | 1.06±0.24 | 1.08±0.53 | 0.89 |
| NKT Cells | 0.674±0.37 | 0.741±0.44 | 0.70 | 1.03±0.36 | 0.720±0.48 | 0.10 |
| NK Cells | 1.01±0.42 | 3.01±5.0 | 0.21 | 1.04±0.37 | 2.66±4.33 | 0.26 |
| Monocytes | 1.03±0.33 | 0.975±0.21 | 0.65 | 1.05±0.31 | 0.91±0.34 | 0.33 |
| Macrophages | 0.977±0.47 | 1.25±0.51 | 0.22 | 0.934±0.36 | 1.29±0.60 | 0.11 |
