# Supplementary material for: Effect of Broccoli Sprouts and Live Attenuated Influenza Virus on Peripheral Blood Natural Killer Cells: A Randomized, Double-Blind Study
Source: PLoS One. 2016 Jan 28;11(1):e0147742. doi: 10.1371/journal.pone.0147742 (PMC4731143; doi:10.1371/journal.pone.0147742)
Supplement: S3 Table — Following NK cell enrichment, NK cells were stimulated with PMA/Ionomycin and blocked with Brefeldin A (only intracellular markers) for 4hrs. Data are presented as mean±std.dev. of percentage of positive cells. N = 22–29. *significantly different from day-1 (p<0.05), tested with paired t test. (DOCX) [file pone.0147742.s006.docx]

S3 Table. LAIV effect on markers of systemic NK cells (percentage of positive cells; regardless of treatment). Following NK cell enrichment, NK cells were stimulated with PMA/Ionomycin and blocked with Brefeldin A (only intracellular markers) for 4hrs. Data are presented as mean±std.dev. of percentage of positive cells. N=22-29. *significantly different from day-1 (p<0.05), tested with paired t test.

| **Marker** | **day-1** | **day2** | **p value**  day2 vs day-1 | **day21** | **p value**  day21 vs day-1 |
| --- | --- | --- | --- | --- | --- |
| CD56 | 80.6±8.7 | 78.5±10 | 0.16 | 75.7±16 | **0.043*** |
| CD16 | 60.8±29 | 74.6±25 | **0.0064*** | 61.0±28 | 0.82 |
| CD314 (NKG2D) | 62.4±11 | 56.7±13 | **0.012*** | 60.7±13 | 0.49 |
| CD158b | 37.9±14 | 35.2±8.2 | 0.39 | 35.6±12.2 | 0.26 |
| CD183 (CXCR3) | 51.1±19 | 42.0±17 | 0.16 | 42.8±17 | **0.048*** |
| IFN-γ | 73.5±17 | 73.1±10 | 0.34 | 71.8±16 | 0.94 |
| IL-4 | 6.99±4.5 | 9.66±11 | 0.42 | 9.62±10 | 0.21 |
| Granzyme B | 56.7±18 | 55.3±20 | 0.25 | 50.9±22 | 0.34 |
