# Supplementary material for: Effect of Broccoli Sprouts and Live Attenuated Influenza Virus on Peripheral Blood Natural Killer Cells: A Randomized, Double-Blind Study
Source: PLoS One. 2016 Jan 28;11(1):e0147742. doi: 10.1371/journal.pone.0147742 (PMC4731143; doi:10.1371/journal.pone.0147742)
Supplement: S4 Table — Following NK cell enrichment, NK cells were stimulated with PMA/Ionomycin and blocked with Brefeldin A (only intracellular markers) for 4hrs. The ratio of day2 or day21 to day-1 are shown. Data are presented as mean±std.dev. N = 9–14. *significantly different (p<0.05), tested with two sample t test. (DOCX) [file pone.0147742.s007.docx]

S4 Table. BSH effect on markers of systemic NK cells (fold induction of day-1). Following NK cell enrichment, NK cells were stimulated with PMA/Ionomycin and blocked with Brefeldin A (only intracellular markers) for 4hrs. The ratio of day2 or day21 to day-1 are shown. Data are presented as mean±std.dev.. N=9-14. *significantly different (p<0.05), tested with two sample t test.

| **Marker** | **Ratio of day2/day-1** | | | **Ratio of day21/day-1** | | |
| --- | --- | --- | --- | --- | --- | --- |
|  | **ASH** | **BSH** | **p value** | **ASH** | **BSH** | **p value** |
| CD56 | 0.780±0.33 | 0.989±0.44 | 0.23 | 0.909±0.77 | 1.00±0.62 | 0.74 |
| CD16 | 9.36±14 | 3.49±3.9 | 0.18 | 5.21±9.6 | 2.14±2.6 | 0.27 |
| CD314 (NKG2D) | 0.820±0.25 | 1.09±0.79 | 0.33 | 0.894±0.27 | 1.18±0.27 | **0.017*** |
| CD158b | 0.731±0.23 | 0.841±0.30 | 0.35 | 0.996±0.68 | 0.884±0.34 | 0.63 |
| CD183 (CXCR3) | 0.651±0.36 | 0.757±0.81 | 0.72 | 0.953±0.81 | 0.872±0.67 | 0.81 |
| IFN-γ | 0.985±0.76 | 1.44±1.2 | 0.29 | 2.21±3.9 | 1.06±0.40 | 0.28 |
| IL-4 | 0.950±0.57 | 1.30±0.71 | 0.20 | 1.35±1.8 | 1.35±1.44 | 0.37 |
| Granzyme B | 0.831±0.32 | 1.13±0.42 | 0.06 | 1.22±1.14 | 1.01±0.39 | 0.50 |
